# Evaluation of *Stylosanthes scabra* Accessions as Forage Source for Ruminants: Growth Performance, Nutritive Value and In Vitro Ruminal Fermentation

**DOI:** 10.3390/ani10111939

**Published:** 2020-10-22

**Authors:** Thamsanqa Doctor Empire Mpanza, Abubeker Hassen, Abiodun Mayowa Akanmu

**Affiliations:** 1 Department of Animal and Wildlife Sciences, University of Pretoria, Private Bag X20, Pretoria Hatfield 0028, South Africa; abubeker.hassen@up.ac.za (A.H.); abiodun.akanmu@up.ac.za (A.M.A.); 2 Agricultural Research Council-Animal Production, Private Bag X2, Irene 0062, South Africa

**Keywords:** forage yield, nutritive value, feeding potential, Shrubby Stylo

## Abstract

**Simple Summary:**

Livestock production under smallholder farmers is limited by scarcity of good quality forage throughout the year. Therefore, there is a need to evaluate alternative forage crops to improve poor quality of the available forage source. *Stylosanths scabra* (Shrubby Stylo) is an important drought tolerant forage legume suitable for seasonally dry environments. It is a shrubby, erect legume with a long tap root system that make the species drought tolerant and able to produce moderate high quality forage under rain fed condition. Hence, *Stylosanthes scabra* is a good candidate species that need to be tested as an alternative forage source for smallholder farmers in tropical and subtropical climatic conditions often prone to drought and frost, owing to their biomass yield and nutritive value.

**Abstract:**

Feed shortage is the main cause of poor production performance in livestock under smallholder farmer’s in South Africa. Therefore, this study evaluated the growth performance, nutritive value and in vitro ruminal fermentation of *Stylosanthes scabra* accessions in climatic condition of Pretoria, South Africa as potential forage sources in order to improve feed quality and subsequently livestock production. *Stylosanthes scabra* accessions were planted in 6 m^2^ plots following a complete randomized block design with three replicates per accession. The plants were allowed to grow to full maturity after which forage was harvested and yield, chemical composition, phenolic compounds, in vitro organic matter digestibility (IVOMD) and in vitro ruminal fermentation characteristic were determined. Forage yield of accessions ranged between 4.3 to 5.3 t ha^−1^ in dry matter (DM), and only twelve accessions were identified to be adaptable over the three-year evaluation under rain-fed. Accessions 140, 9281, 11,252, 11,595, 11,604 and 11,625 were consistent in terms of forage yield over the three years Significant differences among accessions were observed for crude protein (CP), neutral detergent fiber (NDF), IVOMD, metabolizable energy, metabolizable energy yield, gas production, total phenols, total hydrolysable tannins and total condensed tannins. Principal component and clustering analysis showed that accessions 11,255 and 11,625 are distinct in their characteristics as compared to the rest of the accessions, and both accessions are suitable forage source for animals since they contain low NDF with good forage production and crude protein content. However, there is a need for further study to integrate these accessions into the feeding systems in order to improve livestock production.

## 1. Introduction

Livestock rearing by resource-poor smallholder farmers in the sub-Saharan Africa (SSA) region, predominantly depends on poor quality pastures and crop residues especially during the dry season [1,2]. Reliance on poor-quality forage which is often also in limited supply is a major constraint for livestock production [3,4], as the nutrients contained in these forages are not adequate to meet the minimum nutrients requirements for the livestock utilising the natural pastures [5]. This, in turn, often leads to emaciation, reduced reproductive performance and increased livestock mortality rates [4,6].

Forage material from legumes present a good source of protein, energy and mineral supplements for livestock [7,8,9], particularly during the dry season. The evaluation of alternative fodder resources capable of being used as supplementary feed sources is therefore necessary in order to identify superior species and accessions that can survive under specific marginal agro-ecological conditions [10]. However, this area of research is still not well documented for South African subtropical climatic condition which is characterized by drought and accessional frost as in Pretoria.

*Stylosanthes scabra* (Shrubby Stylo), a shrubby perennial legume is native to the tropical areas of South America [11]. Shrubby Stylo grows in tropical and subtropical regions with varying in soil fertility and acidity [12]. Thus, Shrubby Stylo is an important legume for drier regions as it is grows in areas with annual rainfall of 325 mm [12]. *Stylosanthes scabra* grows up to 1.2 m and reported to produce biomass ranging from 1 to 10 and 2 to 7 ton per hectare of dry matter (t ha^−1^ DM) per year as pure pasture or when intercropped with grasses, respectively [13,14]. However, there is paucity of information regarding the performance of *Stylosanthes scabra* in subtropical region of Southern Africa which experience occasional frost in winter. Consequently, there is a need to evaluate the potential of different *S. scabra* accessions in the South African sub-tropical climate, which experience frost during winter. Hence, this study was conducted with an objective of evaluating agronomic performance, nutritive value and in vitro fermentation of *S. scabra* accessions grown in the subtropical climate of South Africa which experiences frost in winter.

## 2. Materials and Methods

### 2.1. Study Site

**Figure 1:**
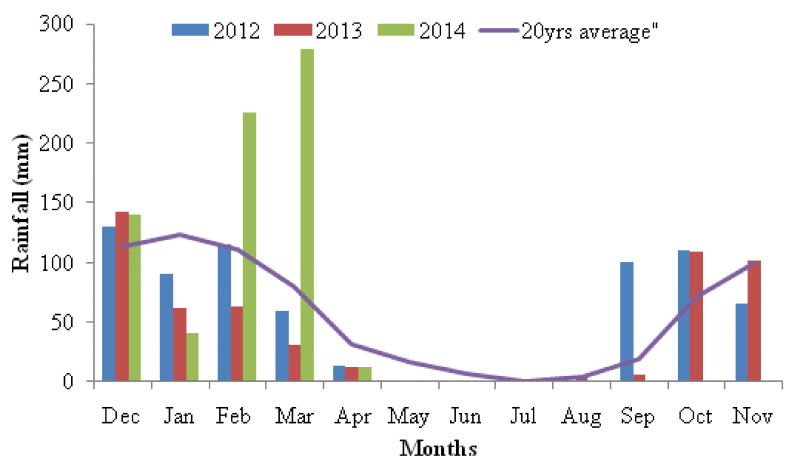
Monthly and 20 years long-term average rainfall data on the study area.

This study was conducted at the University of Pretoria, Hatfield Experimental Farm (25°44’30” S, 28°15’30” E) from 2012 to 2014. The farm is situated at 1370 m above sea level. This area has two seasons, the long dry season extending from March to September and the short wet season extending from October to February. The wet season is warm and humid, while the dry season is cold and sunny between May to July. The study area has average annual rainfall of 674 mm [15]. However, the recorded average rainfall during the study years of 2012, 2013 and 2014 was 683.9 mm, 532.1 mm and 698.5 mm, respectively. Figure 1 shows the monthly average rainfall data and 20 years long-term average rainfall in the study area. Long-term rainfall data (20 years) were obtained from Eendracht weather station in Pretoria (0513314C9), South African. The ethics code for the project is EC085-12.

### 2.2. Experimental Design and Measurements

A total of fifteen *Stylosanthes scabra* accessions were selected based on agro-ecological conditions of the study area in South Africa using the tropical forage website tool [14] (www.tropicalforages.info). *Stylosanthes scabra* accessions used in the study were donated by the International Livestock Research Institute (ILRI) from Ethiopia. Since ILRI donated only 20 g seeds per accession, seedling trays were used first to produce seedlings and when they attained 10 cm long they were transplanted into a 3 × 2 m plot for each accession. Field was prepared to fine tilth and samples of soil were taken at two soil depths and used to determine the soil chemical properties (Table 1). Randomised complete block design (RCBD) was used to arrange treatments (*S. scabra* accessions) with three replicates (three blocks) and there were fifteen *S. scabra* accessions as treatments per block. Each experimental plot had eight rows and each row contained twelve seedlings maintained in a space of 0.25 m and rows within a plot were 0.25 m apart. Plots were maintained at 0.5 m apart within a block, while one meter space was maintained between blocks. In order to reduce competition, weeds were hand removed during the experiment.

#### 2.2.1. Growth Performance

*Stylosanthes scabra* accessions were observed for pest infestation and disease incidence to evaluate their adaptability to the prevailing agro-ecological conditions in Pretoria, Gauteng province of South Africa. In order to assess growth performance, plant height, canopy spread, tillering capacity and plant survival were recorded. Plant height was measured from the ground to the top, while plant canopy diameter was recorded by measuring the horizontal diameter (to nearest 2 cm) of the long and short axis of the canopy on five plants that were randomly selected per plot. In order to record tillering capacity, new sprouts emerging after cutting were counted, in five plants that were selected randomly in each plot [16]. Forage yield was determine by harvesting (whole plot) when plants were at 100% flowering stage by cutting at 5 cm above soil surface. Immediately after cutting fresh weight was recorded and two representative samples of about 100 g per plots were taken, oven dried for 72 h at 60 °C to constant weight and used to determine dry matter (DM) yield.

#### 2.2.2. Nutritive Value

Nutritive value was determined on 12 *Stylosanthes scabra* accessions that were able to produce not less than 3 t h^−1^ DM over three years of evaluation. Forages of these accessions was harvested and representative samples dried in oven as described in Section 2.2.1. After drying, leaves and stems of less than 3 mm were ground to pass 1 mm screen and used for chemical analysis, phenolic compounds, in vitro organic matter digestibility (IVOMD) and in vitro fermentation. For chemical analysis, total N was determined using Kjeldahl methods [17], nitrogen was multiplied by 6.25 to determine crude protein content. Fiber (NDF) content was analysed according to van Soest et al. [18] using ANKOM 200/220 fibre analyser. The procedure of Tilley and Terry [19] was used to determine in vitro organic matter digestibility. Makkar’s [20] procedure was used to determine phenolic compounds that includes total phenols (TP), total hydrolysable tannins (THT) and total condensed tannins (TCT).

#### 2.2.3. In Vitro Ruminal Fermentation

The procedure of Goering and van Soest [21] was followed in conducting ruminal fermentation. However, there were some modification in preparing macro-mineral solution as proposed by Mould et al. [22] where Magnesium sulphate (MgSO_4_.7H_2_O) was replaced by magnesium Chloride (MgCl_2_.6H_2_O) in order to reduce sulphate level in the media. Rumen fluid was collected before morning feeding from two South African Merino sheep (males) fitted with permanent rumen cannula. Sheep were fed alfalfa hay as basal feed with access to fresh water. The details on rumen fluid collection and preparation of substrates for incubation are described in Mpanza [23]. Briefly, twelve accessions referred as treatments were evaluated for in vitro ruminal fermentation. An amount of 400 mg per treatment was weighed into a 120 mL serum bottle in duplicates. Therefore, a total of 26 serum bottles including two blanks without treatment (control) were used per run, and there were three runs conducted separately each taking 96 h. Serum bottles were placed in incubator (120 revolution per minute) set at 39 °C and gas accumulated in head space was recorded in 2, 4, 8, 12, 24, 48, 72 and 96 h by inserting a 23 gauge (0.6 mm) needle attached to a digital data logger as according to Theodorou et al. [24]. Gas cumulative value was calculated as described by Akanmu and Hassen [25].

### 2.3. Data Calculation and Statistical Analysis

Gas pressure was converted into gas volume (GV) using Boyle’s gas law as described by Akanmu and Hassen [25]. Gas volume recorded over time were fitted to non-linear equation as according to Ørskov and McDonald [26].

*Y* = *b* × (1 − e^−*c*t^) (1)

where *Y* is gas volume (mL) at time t, *b* is the insoluble but slowly fermentable fraction (mL), *c* is the rate of gas production per hour, and t is the incubation time. Parameters *b* and *c* were determined by an iterative least square method using NLIN procedure of the SAS [27]. Ørskov and McDonald [26] equation was used to estimate effective gas production (EGP) at a flow rate constant (k) of 0.05 h^−1^.

EGP = b × c/(k + c)(2)

Metabolizable energy (ME) and short chain fatty acid (SCFA) values for different forages of *Stylosanthes scabra* accessions were calculated following the equations of Menke et al. [28] and Menke and Steingass [29], respectively. Metabolizable energy yield (MEY) was calculated as the product of metabolizable energy concentration and average forage yield of each accession, and was expressed in Giga Joules per hectare (GJ ha^−1^).

In an attempt to study the overall relationship between agronomic performance and nutritive value parameters of *S. scabra* accessions, a multivariate principal component analysis (PCA) was performed using the PAST3 software. Data was tested for normality and forage yield data for 2012 and 2014 was not normally distributed, hence for normality the data were square root transformed. In addition, data on growth performance, nutritive value and in vitro ruminal fermentation parameters for forages of *S. scabra* accessions were subjected to analysis of variance using the General Linear Model procedure of the SAS [27], according to the model:

Yi = μ + S_i_ + B_j_ + ε_ij_

where Yi = the response variable (i.e., growth performance, nutritive value and in vitro ruminal fermentation).

μ = Overall mean.
S_i_ = the ith effect of *S. scabra* accessions as treatments (I = 1 to 15).
B_j_ = effect of the jth block (j = 1, 2, 3).
ε_ij_ = random error.

In case of significant differences between the means, Duncan’s new multiple range test (DMRT) was used to separate the means. The level of statistical significance was set at *p* ≤ 0.05.

## 3. Results

### 3.1. General Observation

The establishment was done in 2012 by transplanting seedlings into the plots. This can be responsible to the vigorous growth witnessed from all the accessions. However, few individual plants had some small black insects (pest susceptible), but no noticeable damage was observed on the plants. Since there was no damage observed on plants it was concluded that identification of the insects was not important. All accessions lost their leaves during winter period, and this period was characterised by no rain, low temperatures and occasional frost. However, in spring most accessions sprouted, except accession 11,591.

### 3.2. Multivariate Analysis and General Groupings

**Figure 2:**
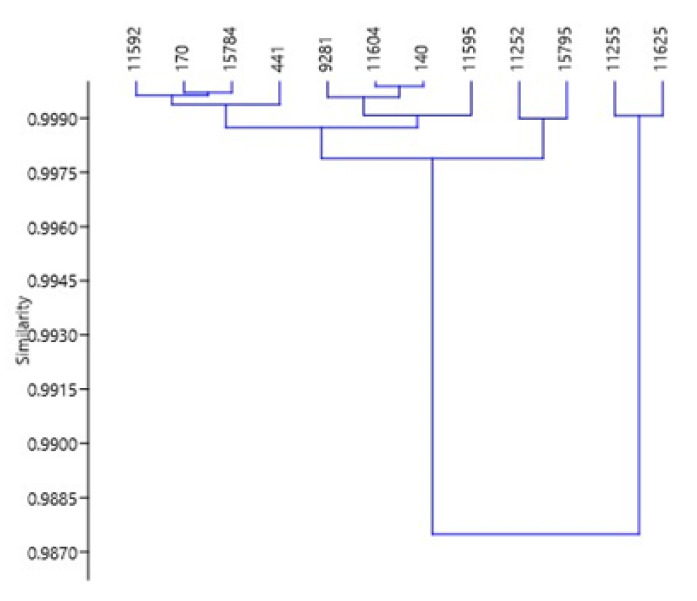
Similarity plot of different *S. scabra* accessions for all parameters measured.

**Figure 3:**
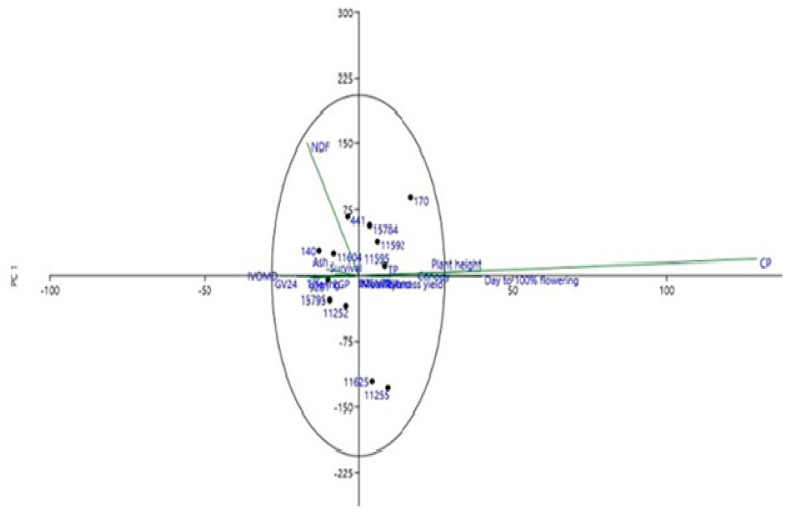
Principal component plot of different *S. scabra* accessions for all parameters measured.

Multivariate analysis shows that principal component 1 (PC1) is responsible for 95.5% of all variations in the study. Using 20% variation as the benchmark, the relationship of the *S. scabra* accessions was divided into positive and negative. *Stylosanthes scabra* accessions 170, 441, 15,784, 11,592 and 11,604 have percentage positive relationship values of 89.3, 66.6, 56.9, 38.4 and 24.4, respectively with PC1 while accessions 11,255, 11,625, 11,252 and 15,795 were negatively correlated with PC1 and had values −127.6, −120, −34 and −28, respectively. The principal component loading in Table 2 explained that the main contributor to all the variations witnessed were NDF and CP, which had 99.04 and 12.06%, respectively. Clustering analysis presented in Figure 2 shows that accessions 11,625 and 11,255 separated distinctly from the rest of accessions mainly due to their NDF values. As observed in Figure 3, these two accessions (11,625 and 11,255) has a lower NDF values as compared to all other accessions.

### 3.3. Growth Performance

Growth performance which include plant height, canopy spread, tillering capacity and survival of the *S. scabra* accessions are shown in Table 3. There was a significant (*p* < 0.05) variation on growth performance between accessions. Plant height ranged from 22.0 to 41.5 cm with accessions 15,795 and 11,591 being the shortest and tallest, respectively. Canopy spread varied significantly (*p* < 0.05), with the diameter ranged from 31.6 cm to 43.6 cm for accessions 9281 and 9268, respectively. There was a significant (*p* < 0.05) variation on tillering capacity between accessions, the highest value (19.9) was recorded for accession 15,795 while the lowest (11.1) was recorded for accession 11,625. Accessions took about 7 to 9 weeks to attain 100% flowering stage (maturity). Winter affected plant survival significantly (*p* < 0.05), where most accessions were above 80%, however, accession 12,555 dropped to 41% survival rate.

Table 4 shows the forage yield of *S. scabra* accessions over the study period. There was a significant variation (*p* < 0.05) amongst accessions during the study period (2012 to 2014). Out of the 15 accessions evaluated, 12 accessions showed persistence in the study area. Although accession 11,591 produced the highest yield (6.7 t ha^−1^ DM) in the establishment year (2012), it however dried up during winter possibly because of low temperatures and frost. The performance of the accessions in terms of forage yield over three years showed that accession 9268 declined by 71% in year three. However, accessions 140, 9281, 11,252, 11,595, 11,604 and 11,625 were consistent in terms of forage yield over the three years. Irrespective of accession, forage yield was significantly lower (*p* < 0.05) in year three (2014).

### 3.4. Chemical Analysis and Phenolic Compounds

Table 5 shows the chemical analysis and phenolic compound for different forages of *S. scabra* accessions adapted in the climatic condition of Pretoria. There was a significant (*p* < 0.05) variation of crude protein between accessions, with the highest value of 230.9 g kg^−1^ DM recorded in accession 170, while accessions 11,625 recorded the lowest value of 189.7 g kg^−1^ DM as shown in the multivariate analysis. The NDF content differed (*p* < 0.05) across the accessions with the highest value of 559.2 g kg^−1^ DM reported in accessions 170 and the lowest value of 345.3 g kg^−1^ DM was recorded for accession 11,255. Thus, the reason NDF was reported as the major source of variation observed in the principal component (PC 1) in Table 2. Phenolic compounds (TP, THT and TCT) varied (*p* < 0.05) among the accessions. Total phenols recorded for accessions ranged between 5.7 and 9.8 g kg^−1^ DM for accession 15,795 and accessions 11,255 and 11,592, respectively. Total hydrolysable tannins recorded in this study ranged between 2.4 and 5.6 g kg^−1^ DM for accession 15,784 and accession 11,595, respectively. However, total hydrolysable tannins was not detected in three of the accessions (140, 9281 and 15,795). On the other hand, the detectable total condensed tannins ranged from 0.5 to 3.1 g kg^−1^ DM in accessions 170 and 11,252, respectively.

### 3.5. Ruminal Fermentation

**Figure 4:**
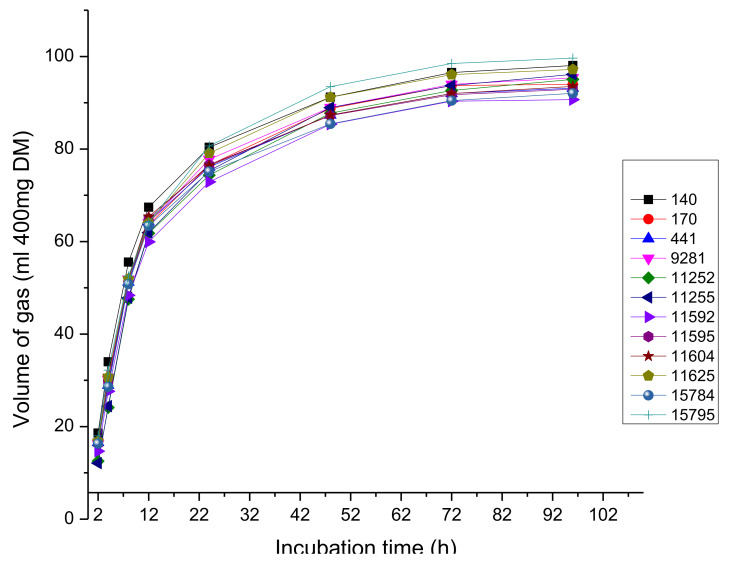
Trend of gas production pattern for different forages of *S. scabra* accessions incubated with rumen fluid in vitro.

The gas production trend over 96 h post incubation for the accessions is shown in Figure 4. Whereas, the kinetics of gas production are presented in Table 6. Variation (*p* < 0.05) among accessions was only observed on the insoluble but slowly fermentable fraction (b).

### 3.6. Feeding Value

In vitro dietary evaluation of the *S. scabra* accessions were assessed by determining IVOMD as described earlier. The noticeable significant (*p* < 0.05) variation among accessions was observed on in vitro organic matter digestibility (IVOMD), metabolizable energy (ME) and metabolizable energy yield (ME yield) Table 7. The ME and SCFA significantly correlated positively with accessions fermentation at various times of incubation, whereas TP significantly correlated negatively with accessions fermentation (Table 8).

## 4. Discussion

Twelve accessions recorded a survival rate of above 85% over study period (three years) which indicate their persistent in the subtropical climate of Pretoria, South Africa. *Stylosanthes scabra* has been reported to be persistent in semi-arid tropical climate of Limpopo province, South Africa [30]. Crop persistent is determined by the longevity of plant and the regeneration of seedlings [31]. The performance of *S. scabra* accessions evaluated in this study was evaluated with respect to re-sprouting after winter and the survival. The re-sprouting of the accessions after winter indicates that plant did not die off during winter and that confirms the results of Hall et al. [32], which indicate the potential of the accessions to adapt to the subtropical climate.

Evaluated *S. scabra* accessions produced above 4 t ha^−1^ DM under rain-fed condition without fertilizer in the establishment year (2012), except accession 15,795 which reported 3.9 t ha^−1^ DM. The forage yield recorded in this study on average were 3.29 and 2.05 times the values of 1.5 and 2.4 t ha^−1^ DM reported by Akinlade et al. [13] and Ciotti et al. [31], respectively. However, the later studies were conducted in savannah zone of Nigeria and sub-humid zone in Argentina, respectively. The high forage yield recorded in the current study could be an attribute of the fact that plots we established by transplanting vigorous seedlings that were produced in the seedling trays. Whereas in the previous studies seeds were used in the establishment of plots, however, *S. scabra* established poorly from seeds, which resulted into low forage yield in the first year of production [13]. Six (140, 9281, 11,252, 11,595, 11,604 and 11,625) of the accessions were observed to be consistent in terms of forage yield over the study years (Table 4).

All the accessions evaluated are rich in crude protein (CP) content ranging from 17.8 to 23.2%, irrespective harvesting the forage at maturity for maximum forage yield. The CP values recorded for *S. scabra* accessions were in the range of values for *S. guianensis*, *Lablab purpureus* and *Vigna unguiculata* but higher than that of *Mucuna pruriens* recorded by Ajayi and Babayemi [33] and Katsande et al. [9], respectively. The crude protein content recorded in this study was in the range of value recommended for lactating dairy cow [34,35]. Two of the accessions (140 and 441) recorded in this study reported crude protein level of 1.34 and 1.38 times the values of 14.7 and 15.0% for the same accessions as reported by Akinlade et al. [13], respectively. The recorded NDF content in this study were in the range of 34.5 to 55.9 %, which is in the range of values for *S. guianensis* and *Lablab purpureus* recorded by Ajayi and Babayemi [33]. The forage with an NDF value below 60% NDF considered good for animals feeding since the value above this reported to depress feed intake owing to poor digestibility [36]. The total phenol content recorded in this study are 0.53 and 0.49 times the values of 14.7 and 15.9 for *S. guianensis* and *Lablab purpureus* as reported by Ajayi and Babayemi [33], respectively. Total hydrolysable tannins recorded in this study is below the upper limit value of 90 g kg^−1^ DM after reduce feed intake and digestion is affected [37]. In addition, total condensed tannin reported in the current study is below 50 g kg^−1^ DM which is the threshold value for animal [38]. The low content of both hydrolysable and condensed tannins on the *S. scabra* accession indicates the safety of the forages to be used as feed source to animals.

Fermentation of forage is influenced by the quality (cell structure, phenolic compounds content) and the potency of rumen fluid [39]. High protein content in forage improves microbial multiplication which influences the extent of fermentation [40]. Gas production kinetics as recorded in Table 6 indicates high degradability of *S. scabra* forages grown in Pretoria. Rate at which forage is degraded in the rumen is an important indicator of the level of digestion [41]. Rate of fermentation observed in this study were in agreement with the values for *Stylosanthes guinensis* recorded by Ajayi and Babayemi [33].

Generally, in vitro gas production are useful in estimating metabolizable energy, organic matter digestibility and short-chain fatty acids of the feed [42]. Values for in vitro organic matter digestibility recorded in this study for different forages of *S. scabra* accessions are higher than the values for varieties of *Avena sativa* L. reported by Kafilzadeh and Heidary [43]. The range of 68.4 to 75.6% of IVOMD recorded in this study for different forages of *S. scabra* accessions indicate good digestibility of forages despite the fact that accessions were harvested at maturity. The calculated value for ME in present study were observed to be higher than those of *Avena sativa* L. and *Vicia sativa* L. as reported by Kafilzadeh and Heidary [43] and Huang et al. [44], respectively. However, they were in the range of values for alfalfa hay as recorded by Abas et al. [45]. Crude protein, fiber content, IVOMD, and ME contents in forage indicates the feed potential of forage [46]. The values of metabolic energy yield reported in this study suggests the potential of the accessions to be included as ingredient in feed formulation for animals, as animal performance also depends on ME content present in the feed [47]. The strong positive correlation between short chain fatty acids and rate of gas production of different forages of *S. scabra* accessions indicate that supplementing poor quality forage with these accessions is likely to improve energy availability to animals.

## 5. Conclusions

The persistence of accessions over the study period (three years) indicates their adaptability to climatic condition of the study area. Accessions 140, 9281, 11,252, 11,595, 11,604 and 11,625 gave consistently higher forage yield over the three years of evaluation. Chemical analysis of the forages shows that these accessions had relatively good protein content, with low fiber (NDF) content. However, of these tested accessions, accessions 11,255 and 11,625 are recommended as good quality forage source since they both have lower NDF with relatively good crude protein content. Consequently, they are identified as a suitable accessions for use as a supplementary forage source to improve poor-quality feed.

## Figures and Tables

**Table 1:** Chemical properties of soil on the study site at Hatfield Experimental Farm, University of Pretoria.

	Soil Depth (cm)	
Properties	0–20	20–40
pH (H_2_O) ^a^	6.39	6.21
Total N (%)	0.02	0.02
Carbon (%)	0.46	0.52
C:N ratio	23.17	23.50
Available P (kg ha^−1^) ^b^	0.63	0.42
Cations ^c^ (cmol kg^−1^)		
Ca	1.94	1.92
Mg	1.05	1.03
K	0.13	0.12
Na	0.04	0.05
CEC ^1^	3.47	3.84
Soil texture ^d^ (%)		
Sand	70.7	66.0
Silt	5.3	6.0
Clay	24.0	28.0

^a^ 1:5 soil:water, ^b^ extractant bray 1, ^c^ ammonium acetate method, ^d^ near infrared determination. ^1^ Cation exchange capacity.

**Table 2:** Principal component (PC) loadings of chemical composition, growth parameters, forage yield, in vitro gas production and digestibility for different forages of *S. scabra* accessions.

Parameters	PC 1 × 100	PC 2 × 100	PC 3 × 100
Plant height	3.50	25.99	50.75
Canopy	−0.19	20.16	5.65
Tillering	−0.61	−10.97	−19.38
Day to 100% flowering	−0.64	26.47	−7.86
Survival	0.97	−5.18	23.09
Mean forage yield	−0.19	0.61	1.50
Ash	4.97	−7.01	70.35
Crude protein	12.06	84.94	−13.37
Neutral detergent fiber	99.04	−10.99	−3.98
Total phenols	−0.59	6.86	3.31
Total tannins	0.22	4.60	4.94
Total condensed tannins	−0.26	5.31	2.42
Insoluble but slowly fermentable fraction	−1.81	−10.25	−5.95
Rate constant of gas production	0.01	−0.009	0.03
Effective gas production	−0.92	−2.35	11.25
Gas volume in 24 h	−0.66	−13.10	−1.50
In vitro organic matter digestibility	−0.28	−16.54	−2.93
Metabolizable energy	0.16	0.50	−0.26
Short chain fatty acid	0.00	−0.11	0.12
Metabolizable energy yield	−0.38	3.98	31.59
Eigenvalue	4662.67	87.57	49.35
% variance	95.534	1.79	1.01

PC 1: The first maximum possible information that could cause variation in data. Whereas PC 2, PC3 are the second and third possible information that could cause variation, respectively.

**Table 3:** Growth performance of *S. scabra* accessions evaluated in climatic condition of, Pretoria.

Accessions (ILRI no) *	Plant Height (cm)	Canopy Spread (cm)	Tillering Capacity	Days to 100% Flowering	% Survival after 3 Years
140	34.8 ^bc^	34.1 ^bcd^	14.1 ^bcd^	56.0 ^cd^	94.8 ^a^
170	43.9 ^a^	39.7 ^ab^	12.3 ^bcd^	63.7 ^abc^	94.5 ^a^
441	41.3 ^ab^	32.9 ^bcd^	13.3 ^bcd^	61.0 ^cd^	86.4 ^a^
9268	39.2 ^abc^	43.6 ^a^	12.3 ^bcd^	53.0 ^d^	85.4 ^a^
9281	35.8 ^bc^	31.6 ^d^	14.9 ^b^	56.3 ^cd^	95.1 ^a^
11,252	33.3 ^c^	39.4 ^abc^	13.5 ^bcd^	61.5 ^cd^	98.1 ^a^
11,255	32.8 ^c^	39.5 ^abc^	14.7 ^bc^	58.5 ^cd^	89.0 ^a^
11,591	41.5 ^ab^	39.9 ^ab^	13.9 ^bcd^	70.7 ^ab^	None
11,592	34.5 ^c^	32.1 ^cd^	13.3 ^bcd^	71.3 ^ab^	88.5 ^a^
11,595	36.8 ^bc^	43.2 ^a^	12.3 ^bcd^	56.3 ^cd^	95.8 ^a^
11,604	34.1 ^c^	36.9 ^abc^	11.6 ^cd^	60.3 ^cd^	95.7 ^a^
11,625	35.4 ^bc^	33.8 ^bcd^	11.1 ^d^	72.0 ^a^	89.9 ^a^
12,555	32.4 ^c^	39.93 ^ab^	14.3 ^bc^	59.3 ^cd^	41.3 ^b^
15,784	34.3 ^c^	38.3 ^abc^	11.7 ^cd^	63.0 ^bc^	93.0 ^a^
15,795	22.0 ^d^	35.3 ^bcd^	19.9 ^a^	56.7 ^cd^	88.3 ^a^
SEM	2.34	2.57	1.05	3.03	5.11

Different letters in superscript within a column indicate that means differ significantly (*p* < 0.05). ILRI: International livestock Research Institute; * ILRI identification numbers; SEM: Standard error of mean.

**Table 4:** Forage yield of different *Stylosanthes scabra* accessions evaluated in climatic condition of Pretoria over three years 2012, 2013 and 2014.

Accessions (ILRI no) *	Forage Yield (t ha^−1^DM)			
	Year-2012	Year-2013	Year-2014	Mean
140	4.2 ^bc^_A_	5.3 ^b^_A_	5.3 ^ab^_A_	4.9
170	4.9 ^bc^_A,B_	5.6 ^ab^_A_	3.6 ^bc^_B_	4.7
441	5.3 ^abc^_A_	5.0 ^b^_A,B_	3.8 ^abc^_B_	3.5
9268	4.4 ^bc^_B_	6.3 ^a^_A_	1.8 ^c^_C_	4.1
9281	4.6 ^bc^_A_	5.0 ^b^_A_	5.4 ^ab^_A_	5.0
11,252	5.4 ^abc^_A_	5.4 ^ab^_A_	4.5 ^abc^_A_	5.1
11,255	5.8 ^ab^_A_	5.4 ^ab^_A_	3.3 ^bc^_B_	4.8
11,591	6.7 ^a^	-	-	-
11,592	5.2 ^abc^_A_	5.6 ^ab^_A_	3.3 ^bc^_B_	4.7
11,595	5.0 ^abc^_A_	5.3 ^b^_A_	6.6 ^a^_A_	5.6
11,604	4.8 ^bc^_A_	5.2 ^b^_A_	5.4 ^ab^_A_	5.1
11,625	4.8 ^bc^_A_	5.3 ^b^_A_	4.5 ^abc^_A_	4.9
12,555	4.4 ^bc^_A_	5.1 ^b^_A_	2.9 ^bc^_B_	4.1
15,784	3.9 ^c^_B_	5.6 ^ab^_A_	4.7 ^ab^_A_	4.7
15,795	4.6 ^bc^_A_	5.3 ^b^_A_	3.3 ^bc^_B_	4.4
SEM	0.14	0.28	0.78	
Year effects	4.9 _A_	5.3 _A_	4.3 _B_	

Different letters in superscript within a column indicate that means differ significantly (*p* < 0.05). Different letters in subscript within a row indicate that means differs significantly (*p* < 0.05). ILRI: International livestock Research Institute; * ILRI identification numbers; SEM: Standard error of mean.

**Table 5:** Chemical analysis and phenolic compounds for different forages of *S. scabra* accessions adapted in climatic condition of Pretoria.

Accessions (ILRI no) *	Chemical Analysis (g kg^−1^ DM)					
	Ash	CP	NDF	TP	THT	TCT
140	92.3	196.5 ^b^	501.7 ^ab^	6.7 ^c^	3.2 ^ab^	nd
170	93.5	230.9 ^a^	559.2 ^a^	6.9 ^c^	4.5 ^ab^	0.5 ^f^
441	97.5	206.7 ^b^	539.2 ^ab^	7.8 ^bc^	3.4 ^ab^	1.9 ^cd^
9281	88.3	195.3 ^b^	469.0 ^ab^	6.8 ^c^	4.2 ^ab^	nd
11,252	86.5	193.4 ^b^	439.9 ^ab^	8.8 ^ab^	3.5 ^ab^	3.1 ^a^
11,255	82.5	198.0 ^b^	345.3 ^b^	9.8 ^a^	3.5 ^ab^	2.3 ^bc^
11,592	84.8	210.9 ^ab^	511.1 ^ab^	9.8 ^a^	4.4 ^ab^	2.3 ^bc^
11,595	86.8	211.7 ^ab^	483.3 ^ab^	8.5 ^b^	5.6 ^a^	2.9 ^ab^
11,604	94.5	197.9 ^b^	498.0 ^ab^	6.9 ^c^	3.0 ^b^	1.0 ^ef^
11,625	82.5	189.7 ^b^	353.9 ^b^	7.8 ^bc^	3.2 ^ab^	1.3 ^de^
15,784	80.0	212.9 ^ab^	529.7 ^ab^	7.2 ^c^	2.4 ^b^	1.8 ^cd^
15,795	76.3	196.3 ^b^	446.7 ^ab^	5.7 ^d^	2.6 ^b^	nd
SEM	7.60	6.87	68.08	0.36	0.75	0.25

Different letters in superscript within a column indicate that means differ significantly (*p* < 0.05). ILRI: International livestock Research Institute; * ILRI identification numbers; CP: crude protein; NDF: neutral detergent fiber; TP: total phenols; THT: total hydrolysable tannins; TCT: total condensed tannins, SEM: Standard error of mean. nd: not detected.

**Table 6:** Gas kinetics parameters for different forages of *S. scabra* accessions adapted in climatic condition of Pretoria.

Accession (ILRI no) *	b (mL 400 mg^−1^)	c (h^−1^)	EGP (mL 400 mg^−1^)
140	90.2 ^ab^	0.097	59.0
170	88.8 ^ab^	0.091	56.9
441	88.6 ^ab^	0.096	57.8
9281	90.2 ^ab^	0.093	58.1
11,252	89.2 ^ab^	0.086	59.0
11,255	90.8 ^ab^	0.086	59.8
11,592	86.2 ^b^	0.087	54.5
11,595	82.0 ^b^	0.099	58.9
11,604	87.8 ^ab^	0.098	57.9
11,625	91.4 ^a^	0.088	57.8
15,784	87.3 ^ab^	0.097	57.2
15,795	91.7 ^a^	0.084	56.7
SEM	3.79	0.0099	1.76

Different letters in superscript indicate that means differ significantly (*p* < 0.05). ILRI: International livestock Research Institute; * ILRI identification numbers; b: insoluble but slowly fermentable fraction; c: rate of fermentation; EGP: effective gas production; SEM: Standard error of mean.

**Table 7:** Gas production, in vitro organic matter digestibility, metabolizable energy, short chain fatty acid and metabolizable energy yield for different forages of *S. scabra* accessions adapted in climatic condition of Pretoria.

Accessions (ILRI no) *	In Vitro Parameters				
	GV_24h_ (mL 400 mg^−1^)	IVOMD (% DM)	ME (MJ kg^−1^ DM)	SCFA (µmol g^−1^ DM)	ME Yield (GJ ha^−1^)
140	80.4	75.6 ^a^	9.9 ^ab^	0.9	48.9 ^ab^
170	76.5	72.3 ^abc^	10.2 ^a^	0.9	47.1 ^ab^
441	76.1	69.7 ^bc^	9.8 ^ab^	0.8	45.8 ^ab^
9281	77.8	76.5 ^a^	9.7 ^ab^	0.9	48.9 ^ab^
11,252	75.6	69.8 ^bc^	9.5 ^b^	0.8	48.4 ^ab^
11,255	74.9	71.6 ^abc^	9.5 ^b^	0.8	45.9 ^ab^
11,592	72.9	67.4 ^c^	9.6 ^ab^	0.8	45.4 ^ab^
11,595	76.6	68.4 ^c^	9.9 ^ab^	0.9	55.5 ^a^
11,604	76.4	71.8 ^abc^	9.6 ^ab^	0.9	48.5 ^ab^
11,625	79.1	71.1 ^abc^	9.7 ^ab^	0.9	49.7 ^ab^
15,784	75.1	71.4 ^abc^	9.8 ^ab^	0.8	44.7 ^ab^
15,795	80.7	74.7 ^ab^	9.9 ^ab^	0.9	42.6 ^b^
SEM	2.45	1.62	0.20	0.03	3.15

Different letters superscript indicate that means differ significantly (*p* < 0.05). ILRI: International Livestock Research Institute; * ILRI identification numbers; GV: gas volume; IVOMD: in vitro organic matter digestibility; ME: metabolizable energy; SCFA: short chain fatty acid; ME yield: metabolizable energy yield; SEM: standard error of mean.

**Table 8:** Pearson correlation coefficients among chemical analysis, phenolic compounds and gas kinetics.

	CP	ME	SCFA	IVOMD	NDF	Ash	TP	THT	TCT
2 h	−0.088	0.583 **	0.804 **	0.217	0.134	0.258	−0.422 *	0.09	−0.455
4 h	−0.058	0.651 **	0.858 **	0.248	0.151	0.355	−0.405 *	0.16	−0.422
8 h	−0.056	0.684 **	0.899 **	0.291	0.166	0.401	−0.388	0.156	−0.373
12 h	−0.041	0.679 **	0.879 **	0.295	0.171	0.448 *	−0.355	0.104	−0.273
24 h	−0.239	0.611 **	0.994 **	0.453 *	0.012	0.214	−0.498 *	0.051	−0.302
48 h	−0.261	0.509 *	0.891 **	0.505 *	−0.071	0.037	−0.453 *	−0.058	−0.198
72 h	−0.171	0.507 *	0.799 **	0.528 **	−0.017	−0.009	−0.457 *	−0.089	−0.195
96 h	−0.292	0.23	0.583 **	0.599 **	−0.152	−0.164	−0.434 *	−0.178	−0.055
b	−0.149	−0.363	−0.29	0.300	−0.183	−0.249	−0.038	−0.31	0.304
c	0.069	0.567 **	0.629 **	0.005	0.210	0.564 **	−0.122	0.201	−0.203
EGP	−0.108	0.277	0.458 *	0.379	0.056	0.439 *	−0.084	−0.157	0.237

* = significant at *p* < 0.05; ** = significant at *p* < 0.01. CP: crude protein; ME: metabolizable energy; SCFA: short chain fatty acid; IVOMD: in vitro organic matter digestibility; NDF: neutral detergent fibre; TP: total phenols; THT: total hydrolysable tannins; TCT: total condensed tannins; 2 h: two hour gas production; 24 h: twenty four hour gas production; 48 h: Forty eight hour gas production; 96 Ninety six hour gas production; b: insoluble but slowly fermentable fraction; c: rate of fermentation; EGP: effective gas production.
